# Supplementary material for: Targeting mir128-3p alleviates myocardial insulin resistance and prevents ischemia-induced heart failure
Source: eLife. 2020 Mar 30;9:e54298. doi: 10.7554/eLife.54298 (PMC7124275; doi:10.7554/eLife.54298)
Supplement: Supplementary file 1. [file elife-54298-supp1.docx]

| **Key Resources Table** | | | | |
| --- | --- | --- | --- | --- |
| **Reagent type (species) or resource** | **Designation** | **Source or reference** | **Identifiers** | **Additional information** |
| strain, strain background (male *Mus musculus*) | C57BL/6N | Envigo UK | 032080 | 12-weeks-old  RRID:IMSR_JAX:005304 |
| strain, strain background (male *Rattus norvegicus*) | Sprague-Dawley | Envigo UK | 0670 | 6-week-old  RRID:RGD_737903 |
| strain, strain background (male *Mus musculus*) | *Mapk7*-cko, C57BL/6J background | Liu et al., 2017 |  | *Mapk7^flox^* crossed with Tg(*Myhca-cre*) mice  RRID:MGI:3042043 |
| genetic reagent (*Homo sapiens*) | *Irs1* 3´UTR | This paper | 500 bp region from the 3’ UTR *Irs1* gene. | Cloned from human iPSC-CM genomic DNA using the following primers:  F:CGCTAGCTAGCATGACCTCAGCAAATCCTCTTCT  R:CGCTAGTCGACGTATACCTCCATCCCACATCC  Wei Liu lab, University of Manchester. |
| genetic reagent (*Homo sapiens*) | *R3hdm1* promoter | This paper | 1.5 kbp region downstream of first *R3hdm1* transcription start site. | Cloned from human iPSC-CM genomic DNA using the following primers:  F:TCAGTCGAGCTCACAGTTCAAACTGACCACGCGA  R:CGCTAGATATCCCTGCCTCCTTCTCCGTTAGC  Wei Liu lab, University of Manchester. |
| genetic reagent (*Homo sapiens*) | *mir128-1* promoter | This paper | 1.5 kbp region downstream of second *R3hdm1* transcription start site. | Cloned from human iPSC-CM genomic DNA using the following primers:  F:TCAGTCGGTACCTGCATTTTAGCTTGTCCATCTGTT  R:TCAGTCCTCGAGTGATGCTCTGCATTGCTCCT  Wei Liu lab, University of Manchester. |
| genetic reagent (*Homo sapiens*) | *mir128-3p* reporter | This paper | Sigma | Sequence described in Methods and Materials section –Luciferase reporter assay.  Wei Liu lab, University of Manchester. |
| genetic reagent (*Homo sapiens*) | MAPK7 coding sequence | Liu et al., 2017 |  | From the pcDNA5-Flag-Mapk7 plasmid kindly provided by the Cathy Tournier lab. |
| genetic reagent (*Homo sapiens*) | CREB1 coding sequence | Addgene | ID #82203 | RRID:Addgene_82203 |
| genetic reagent (*Homo sapiens*) | CEBPβ coding sequence | Addgene | ID #15738 | RRID:Addgene_15738 |
| cell line (*Rattus norvegicus*) | H9C2 | ATCC | CRL-1446 | RRID:CVCL_0286 |
| cell line (*Homo sapiens*) | HEK293T | ATCC | CRL-3216 | RRID:CVCL_0063 |
| transfected construct (*Aequorea victoria*) | pSSV9-*TnT-Gfp* | Werfel et al., 2014 |  | Used to produce AAV9 vectors for overexpression. |
| transfected construct (*Adeno-associated dependoparvovirus*) | pDGΔVP | Werfel et al., 2014 |  | Used to produce AAV9 vectors. |
| transfected construct (*Adeno-associated dependoparvovirus*) | pAAV2-9 Rep-Cap plasmid (p5E18-VD2/9) | Werfel et al., 2014 |  | Used to produce AAV9 vectors. |
| transfected construct (*Aequorea victoria*) | pSSV9-*TnT-H1-inteGFP* | Werfel et al., 2014 |  | Used to produce AAV9 vector for antimiR128 TuD. |
| transfected construct (*Mus musculus*) | pLenti-*myc-Slc2a4-mCherry* | Addgene | ID #64049 | RRID:Addgene_64049 |
| transfected construct (*Homo sapiens*) | pGL3-Basic | Promega | E1751 |  |
| transfected construct (*Homo sapiens*) | pmiRGlo | Promega | E1330 |  |
| biological sample (*Homo sapiens*) | Human myocardial protein extracts | Asterand |  |  |
| antibody | sheep polyclonal anti-DIG AP FAB fragments | Sigma | 11093274910 | IS 1:800  RRID:AB_2734716 |
| antibody | Mouse monoclonal anti-α-Actinin | Sigma | A7811 | IF 1:100  RRID:AB_476766 |
| antibody | Rabbit polyclonal anti-SLC2A4 | Santa Cruz Biotechnology | sc-7938 | WB 1:500  IF 1:100  RRID:AB_2254987 |
| antibody | Rabbit polyclonal anti-pMAPK7-Thr218/Tyr220 | Cell Signaling | 3371 | WB 1:500  RRID:AB_2140424 |
| antibody | Rabbit polyclonal anti- MAPK7 | Cell Signaling | 3372 | WB 1:1000  RRID:AB_330491 |
| antibody | Rabbit polyclonal anti- SLC2A1 | Santa Cruz Biotechnology | sc-7903 | WB 1:1000  RRID:AB_2190936 |
| antibody | Mouse monoclonal anti- SLC2A4 | Thermo Fisher Scientific | MA183191 | WB 1:1000  RRID:AB_2191429 |
| antibody | Rabbit polyclonal anti-IR | Cell Signaling | 3025 | WB 1:1000  RRID:AB_2280448 |
| antibody | Rabbit polyclonal anti-pIRS1-Tyr608 | Millipore | 09-432 | WB 1:1000  RRID:AB_1163457 |
| antibody | Rabbit polyclonal anti-IRS1 | Cell Signaling | 2382 | WB 1:1000  RRID:AB_330333 |
| antibody | Rabbit polyclonal anti-pCREB1-Ser133 | Cell Signaling | 9198 | WB 1:1000  IF 1:100  RRID:AB_2561044 |
| antibody | Rabbit polyclonal anti-CREB1 | Cell Signaling | 9197 | WB 1:1000  RRID:AB_331277 |
| antibody | Rabbit polyclonal anti-CEBPβ | Cell Signaling | 3087 | WB 1:500  RRID:AB_2078052 |
| antibody | Mouse monoclonal anti-CEBPβ | Santa Cruz Biotechnology | sc-7962 | WB 1:500  IF 1:50  RRID:AB_626772 |
| antibody | Rabbit polyclonal anti-pAKT1-Ser473 | Cell Signaling | 9271 | WB 1:1000  RRID:AB_329825 |
| antibody | Rabbit polyclonal anti-AKT | Cell Signaling | 9272 | WB 1:1000  RRID:AB_329827 |
| antibody | Rabbit polyclonal anti-pMEF2A-S488 | Cell Signaling | 9737 | WB 1:1000  RRID:AB_2297576 |
| antibody | Rabbit polyclonal anti-MEF2A | Abcam | ab32866 | WB 1:1000  RRID:AB_776267 |
| antibody | Rabbit polyclonal anti-MEF2C | Abcam | ab64644 | WB 1:1000  RRID:AB_2142861 |
| antibody | Rabbit polyclonal anti-pMEF2C | Sigma | SAB4504712 | WB 1:1000  RRID:AB_2827764 |
| antibody | Rabbit polyclonal anti-cleaved caspase 3 | Cell Signaling | 9661 | WB 1:500  RRID:AB_2341188 |
| antibody | Rabbit polyclonal anti-caspase 3 | Cell Signaling | 9662 | WB 1:1000  RRID:AB_331439 |
| antibody | Mouse polyclonal anti-GΒ | Santa Cruz Biotechnology | sc-166123 | WB 1:500  RRID:AB_2109632 |
| antibody | Horse anti-mouse-HRP | Cell Signaling | 7076 | WB 1:2000  RRID:AB_330924 |
| antibody | Horse anti-rabbit-HRP | Cell Signaling | 7074 | WB 1:2000  RRID:AB_2099233 |
| antibody | Rabbit IgG | Cell Signaling | 2729 | ChIP 1:50  RRID:AB_1031062 |
| antibody | Donkey anti-rabbit-Alexa-488 | Jackson ImmunoResearch | 711-545-152 | IF 1:500  RRID:AB_2313584 |
| antibody | Donkey anti-mouse-Alexa-594 | Jackson ImmunoResearch | 715-585-150 | IF 1:500  RRID:AB_2340854 |
| antibody | Rabbit polyclonal anti-MYC | Cell Signaling | 2272 | IF 1:400  RRID:AB_10692100 b |
| sequence-based reagent | *antimir128-3p* TuD | This paper | Sigma | Sequence described in Methods and Materials section – AntimiR-128 tough decoy (TuD) design.  Wei Liu lab, University of Manchester. |
| sequence-based reagent | antimir Control TuD | This paper | Sigma | Sequence described in Methods and Materials section – AntimiR-128 tough decoy (TuD) design.  Wei Liu lab, University of Manchester. |
| sequence-based reagent | miRCURY LNA miRNA Detection Probes | Qiagen | 339111 | Sequence not available. |
| sequence-based reagent | siScramble | Sigma |  | AGGUAGUGUAAUCGCCUUGtt |
| sequence-based reagent | si*Mapk7* | Sigma |  | AAAGGGUGCGAGCCUAUAUtt |
| sequence-based reagent | *Mapk7* SignalSilencer | Cell Signaling | 7301 | Sequence not available. |
| sequence-based reagent | *Creb1* Silencer Select | Ambion | s135439 | GAGAAUGUCGUAGAAAGAAtt |
| sequence-based reagent | *Cebpb* Silencer Select | Ambion | s127566 | GCAAGAAGCCGUCCGACUAtt |
| sequence-based reagent | *Irs1* Silencer Select | Ambion | s129870 | GGGUGAACCUAAGUCCCAAtt |
| sequence-based reagent | *mir128-3p* precursor | Ambion | PM114746 | Sequence not available. |
| sequence-based reagent | *mir128-3p* inhibitor | Ambion | AM11746 | Sequence not available. |
| sequence-based reagent | *Pri-mir128-1* primers | Applied Biosystems | Mm03306255_pri | Sequence not available. |
| sequence-based reagent | *Pri-mir128-2* primers | Applied Biosystems | Mm03307278_pri | Sequence not available. |
| sequence-based reagent | *mir128-3p* TaqMan Advanced miRNA assay | Applied Biosystems | 477892_mir | Sequence not available. |
| sequence-based reagent | *mir191-5p* TaqMan Advanced miRNA assay | Applied Biosystems | 477952_mir | Sequence not available. |
| peptide, recombinant protein | Superscript II Reverse Transcriptase | Invitrogen | 18064 |  |
| commercial assay or kit | Ultra-Sensitive Mouse Cardiac Troponin-I Elisa | Life Diagnostics | CTNI-1-US |  |
| commercial assay or kit | LDH activity assay | Sigma | MAK066 |  |
| commercial assay or kit | miRCURY LNA miRNA ISH Buffer Set | Qiagen | 339450 |  |
| commercial assay or kit | Periodic Acid-Schiff (PAS) Staining System | Sigma Aldrich | 395B |  |
| commercial assay or kit | In Situ Cell Death Detection Kit | Roche | 11684795910 |  |
| commercial assay or kit | SYBR Select PCR Master Mix | Applied Biosystems | 4472908 |  |
| commercial assay or kit | TaqMan Gene Expression Master Mix | Applied Biosystems | 4369016 |  |
| commercial assay or kit | TaqMan Advanced miRNA cDNA Synthesis Kit | Applied Biosystems | A28007 |  |
| commercial assay or kit | TaqMan Fast Advanced Master Mix | Applied Biosystems | 4444557 |  |
| commercial assay or kit | SimpleChip Plus Enzymatic ChIP Kit | Cell Signaling | 9004 |  |
| commercial assay or kit | Protein G sepharose | Sigma | P3296 |  |
| commercial assay or kit | Glucose Uptake-Glo Assay | Promega | J1342 |  |
| commercial assay or kit | ATP Bioluminiscent Assay Kit | Sigma-Aldrich | FLAA |  |
| commercial assay or kit | Dual-Luciferase Reporter Assay System | Promega | E1980 |  |
| commercial assay or kit | QuickChange Site-Directed Mutagenesis Kit | Agilent Technologies | 200519-5 |  |
| software, algorithm | ImageJ | NIH | https://imagej.nih.gov/ij/download.html | RRID:SCR_003070 |
| software, algorithm | GraphPad Prism 8 | GraphPad Software | https://www.graphpad.com/scientific-software/prism/ | RRID:SCR_002798 |
| software, algorithm | TargetScan | Whitehead Institute for Biomedical Research | http://www.targetscan.org/vert_72/ | RRID:SCR_010845 |
| software, algorithm | miRBase | University of Manchester | http://www.mirbase.org/ | RRID:SCR_003152 |
